# Supplementary material for: Nutrients and Caloric Intake Associated with Fruits, Vegetables, and Legumes in the Elderly European Population
Source: Nutrients. 2020 Sep 9;12(9):2746. doi: 10.3390/nu12092746 (PMC7551243; doi:10.3390/nu12092746)
Supplement: Supplementary file 1 [file nutrients-12-02746-s001.pdf]

Supplementary Tables

Table S1. EFSA surveys sourced for this study

List of food surveys (country, year, and name of survey) included in the EFSA database (65-74 years).

	Country, A-Z	Year	Survey	Questionnaire
EFSA	Austria	2010	Austrian Study on Nutritional Status 2010-12 – Adults	24-hours dietary recall
	Belgium	2004	Diet National 2004	24-hours dietary recall
	Denmark	2000	Danish Dietary Survey	Food record
	Denmark	2005	The Danish National Dietary survey 2005-2008	Food record
	Estonia	2013	National Dietary Survey among 11-74 years old individuals in Estonia	Food record, 24-hours dietary recall
	Finland	2007	National Findiet Surveys	48-hours dietary recall
	Finland	2012	National FINDIET 2012 Survey	48-hours dietary recall
	France	2007	Individual and national study on food consumption 2	Food record, 24-hours dietary recall
	France	2014	The French national dietary survey (INCA3, 2014-2015)	Food record, 24-hours dietary recall
	Germany	2007	National Nutrition Survey II	24-hours dietary recall
	Hungary	2003	National Repr Surv	Food record
	Ireland	2008	National Adult Nutrition Survey	Food record
	Italy	2005	Italian National Food Consumption Survey INRAN-SCAI 2005-06	Food record
	Latvia	2011	Latvian National Dietary survey	Food record, 24-hours dietary recall
	Netherlands	2007	Dutch National food consumption survey 2007 – 2010	Food record, 24-hours dietary recall
	Netherlands	2010	Dutch National Food Consumption Surveys - Older Adults	Food record, 24-hours dietary recall
	Portugal	2015	National Food, Nutrition and Physical Activity Survey of the Portuguese general population	Food record, 24-hours dietary recall
	Romania	2012	Dieta Pilot Adults	Food record
	Spain	2013	Spanish National dietary survey in adults, elderly and pregnant women	Food record, 24-hours dietary recall
	Sweden	2010	Swedish National Dietary Survey - Riksmaten adults 2010-11	Web-based dietary record
	UK	2008-09	National Diet and Nutrition Survey - Years 1-3	Food record

Table S2. **Elderly mean consumption of fruit, vegetables, legumes**

Data taken from the EFSA database (65-74 years), which correspond to the surveys listed in Table S1. Fruit and fruit products (excludes juice); vegetables and vegetable products (includes podded vegetable legumes, soya beans, and fresh herbs, excludes vegetable juice); legumes (fresh and dried legumes, excluding podded vegetable legumes, including dried herbs); fruit and vegetable juice as grams/person/day (g/p/d).

			Fruit	Vegetables	Legumes	Juices	Total
**Country**	**Year**	*N*	g/p/d	g/p/d	g/p/d	g/p/d	g/p/d
Austria	2010	*67*	150.8	81.5	1.5	53.4	287.2
Belgium	2004	*511*	162.2	136.2	5.4	38.4	342.2
Denmark	2000	*309*	196.5	146.3	7.1	77.4	427.3
Denmark	2005	*274*	211.7	128.9	6.9	75.5	423.0
Estonia	2013	*524*	222.4	150.7	5.3	49.0	427.4
Finland	2007	*463*	179.6	112.9	5.2	90.9	388.6
Finland	2012	*413*	197.3	139.4	4.1	99.9	440.7
France	2007	*264*	219.7	186.7	21.6	39.2	467.2
France	2014	*384*	213.7	303.5	8.6	49.5	575.3
Germany	2007	*2006*	229.3	105.4	6.9	139.9	481.5
Hungary	2003	*206*	210.3	166.2	16.6	11.6	404.7
Ireland	2008	*149*	125.5	142.7	12.7	42.4	323.3
Italy	2005	*290*	256.5	248.9	12.2	21.0	538.6
Latvia	2011	*300*	163.4	186.7	8.8	40.5	399.4
Netherlands	2007	*173*	156.5	141.2	3.1	64.6	365.4
Netherlands	2010	*289*	166.7	174.6	4.1	75.0	420.4
Portugal	2015	*509*	161.4	142.8	13.2	16.9	334.3
Romania	2012	*83*	170.2	382.9	11.6	2.8	567.5
Spain	2013	*264*	233.8	156.0	11.6	37.8	439.2
Sweden	2010	*295*	173.2	74.2	5.8	59.0	312.2
UK	2008-09	*166*	128.5	155.9	26.8	46.3	357.5
Mean of all EFSA studies			187.1	164.9	9.5	53.9	415.4

Table S3. EFSA database estimated daily availability of fiber and nutrients from total fruit, vegetables, and legumes (males)

Estimated percentage contributions of daily elderly intake of fiber and nutrients from total fruit, vegetables, fruit and vegetable juice, and legumes (determined and summed from the mean consumption of each individual product at level 7 in the EFSA database (65-74 years) and corresponding nutrient data in food conversion databases *(49) (50)*) to meeting U.S. Recommended Dietary Allowances (RDAs) or Adequate Intakes (AIs *): males aged >70 years.

Country EFSA database	Year	G cluster	Consumption total F+V+J+L mean (g/p/d)	Energy (kcal/d)	Dietary fiber (g/d) *	Potassium (mg/d) *	Calcium (mg/d)*	Magnesium (mg/d)	Phosphorus (mg/d)	Iron (mg/d)	Copper (mg/d)	Zinc (mg/d)	Manganese (mg/d)*	Selenium (µg/d)	RAE (µg/d)	Vitamin E (mg/d)	Vitamin K1 (µg/d)*	Thiamin (mg/d)	Riboflavin (mg/d)	Niacin (mg/d)	Vitamin B6 (mg/d)	Folate (µg/d)	Pantothenate (mg/d)*	Vitamin C (mg/d)
U.S. RDAs (AI) males >70 y*					*30**	*4700**	*1200**	*420*	*700*	*8*	*0.9*	*11*	*2.3**	*55*	*900*	*15*	*120**	*1.2*	*1.3*	*16*	*1.7*	*400*	*5**	*90*
Austria	2010	G08	287.2	164.3	10%	12%	4%	7%	8%	13%	13%	3%	20%	2%	7%	5%	43%	19%	6%	6%	13%	13%	10%	77%
Belgium	2004	G11	342.2	165.2	15%	14%	5%	8%	11%	14%	18%	4%	17%	3%	13%	7%	41%	26%	9%	9%	14%	19%	13%	66%
Denmark	2000	G15	427.3	220.5	17%	18%	6%	11%	13%	19%	20%	4%	21%	2%	20%	9%	32%	38%	9%	11%	18%	19%	16%	87%
Denmark	2005	G15	423.0	253.3	16%	19%	6%	11%	14%	19%	20%	5%	24%	2%	19%	7%	32%	31%	9%	11%	18%	19%	17%	88%
Estonia	2013	G10	427.4	219.6	18%	17%	7%	10%	12%	21%	20%	5%	22%	2%	15%	6%	25%	25%	9%	9%	18%	17%	15%	79%
Finland	2007	G07	388.6	182.7	15%	14%	6%	9%	12%	16%	17%	4%	19%	1%	11%	6%	25%	35%	8%	9%	14%	17%	16%	**104%**
Finland	2012	G07	440.7	199.2	16%	15%	6%	10%	12%	16%	18%	4%	22%	2%	18%	8%	31%	38%	9%	10%	16%	22%	18%	**108%**
France	2007	G07	467.2	241.7	27%	20%	10%	14%	21%	47%	27%	9%	33%	8%	21%	8%	41%	31%	11%	12%	17%	30%	21%	79%
France	2014	G07	575.3	240.7	26%	24%	12%	15%	21%	30%	30%	8%	29%	5%	23%	13%	63%	38%	13%	14%	24%	37%	22%	98%
Germany	2007	G08	481.5	390.0	16%	26%	8%	15%	18%	35%	23%	5%	38%	3%	11%	7%	36%	28%	11%	13%	20%	17%	18%	**121%**
Hungary	2003	G15	404.7	171.8	21%	14%	7%	10%	15%	23%	21%	6%	19%	2%	11%	6%	50%	33%	10%	9%	19%	18%	14%	72%
Ireland	2008	G15	323.3	142.6	14%	14%	6%	9%	13%	16%	18%	5%	18%	3%	21%	8%	40%	30%	8%	10%	16%	20%	15%	68%
Italy	2005	G10	538.6	197.3	23%	30%	11%	20%	23%	34%	44%	9%	28%	6%	34%	23%	84%	62%	18%	22%	25%	36%	25%	**122%**
Latvia	2011	G10	399.4	178.2	21%	16%	9%	11%	13%	31%	19%	6%	30%	2%	21%	7%	38%	24%	8%	9%	16%	25%	16%	89%
Netherlands	2007	G11	365.4	154.5	15%	14%	6%	9%	12%	16%	18%	4%	20%	3%	14%	8%	73%	32%	9%	9%	15%	25%	16%	83%
Netherlands	2010	G11	420.4	186.3	18%	17%	8%	11%	14%	21%	21%	5%	22%	3%	17%	9%	75%	33%	10%	10%	18%	28%	18%	92%
Portugal	2015	G15	334.3	142.3	14%	15%	7%	11%	14%	21%	24%	6%	22%	2%	17%	10%	23%	30%	8%	9%	16%	20%	14%	67%
Romania	2012	G15	567.5	221.8	26%	25%	10%	14%	20%	27%	32%	8%	27%	4%	30%	14%	41%	34%	11%	15%	23%	28%	21%	89%
Spain	2013	G08	439.2	186.5	20%	17%	7%	12%	16%	22%	29%	6%	26%	5%	12%	10%	48%	39%	11%	12%	18%	27%	18%	104%
Sweden	2010	G15	312.2	163.7	12%	12%	4%	8%	10%	13%	16%	3%	16%	1%	10%	6%	18%	28%	7%	8%	13%	15%	12%	74%
United Kingdom	2008	G07	357.5	171.8	19%	17%	6%	11%	17%	22%	25%	7%	31%	12%	18%	10%	48%	32%	9%	12%	21%	23%	18%	72%
Mean			*415.4*	*199.7*	*18%*	*18%*	*7%*	*11%*	*15%*	*23%*	*22%*	*6%*	*24%*	*4%*	*17%*	*9%*	*43%*	*33%*	*10%*	*11%*	*18%*	*23%*	*17%*	*88%*

Table S4. EFSA database estimated daily availability of fiber and nutrients from total fruit, vegetables, and legumes (females)

Estimated percentage contributions of daily elderly intake of fiber and nutrients from total fruit, vegetables, fruit and vegetable juice, and legumes (determined and summed from the mean consumption of each individual product at level 7 in the EFSA database (65-74 years) and corresponding nutrient data in food conversion databases *(49) (50)*) to meeting U.S. Recommended Dietary Allowances (RDAs) or Adequate Intakes (AIs *): females aged >70 years.

Country EFSA database	Year	G cluster	Consumption total F+V+J+L mean (g/p/d)	Energy (kcal/d)	Dietary fiber (g/d) *	Potassium (mg/d) *	Calcium (mg/d)*	Magnesium (mg/d)	Phosphorus (mg/d)	Iron (mg/d)	Copper (mg/d)	Zinc (mg/d)	Manganese (mg/d)*	Selenium (µg/d)	RAE (µg/d)	Vitamin E (mg/d)	Vitamin K1 (µg/d)*	Thiamin (mg/d)	Riboflavin (mg/d)	Niacin (mg/d)	Vitamin B6 (mg/d)	Folate (µg/d)	Pantothenate (mg/d)*	Vitamin C (mg/d)
U.S. RDAs (AI) Females >70 y*					*21**	*4700**	*1200**	*320*	*700*	*8*	*0.9*	*8*	*1.8**	*55*	*700*	*15*	*90**	*1.1*	*1.1*	*14*	*1.5*	*400*	*5**	*75*
Austria	2010	G08	287.2	164.3	14%	12%	4%	10%	8%	13%	13%	4%	25%	2%	9%	5%	58%	21%	7%	7%	15%	13%	10%	92%
Belgium	2004	G11	342.2	165.2	21%	14%	5%	11%	11%	14%	18%	6%	22%	3%	17%	7%	54%	29%	11%	10%	16%	19%	13%	79%
Denmark	2000	G15	427.3	220.5	24%	18%	6%	14%	13%	19%	20%	6%	27%	2%	26%	9%	42%	42%	10%	13%	20%	19%	16%	104%
Denmark	2005	G15	423.0	253.3	22%	19%	6%	15%	14%	19%	20%	6%	31%	2%	24%	7%	43%	34%	11%	12%	20%	19%	17%	106%
Estonia	2013	G10	427.4	219.6	25%	17%	7%	13%	12%	21%	20%	7%	29%	2%	20%	6%	34%	28%	11%	10%	21%	17%	15%	95%
Finland	2007	G07	388.6	182.7	21%	14%	6%	12%	12%	16%	17%	6%	24%	1%	15%	6%	33%	38%	10%	10%	16%	17%	16%	**125%**
Finland	2012	G07	440.7	199.2	23%	15%	6%	13%	12%	16%	18%	6%	28%	2%	24%	8%	41%	41%	11%	12%	18%	22%	18%	**130%**
France	2007	G07	467.2	241.7	38%	20%	10%	18%	21%	47%	27%	12%	42%	8%	27%	8%	55%	34%	13%	13%	19%	30%	21%	95%
France	2014	G07	575.3	240.7	37%	24%	12%	19%	21%	30%	30%	11%	38%	5%	29%	13%	84%	42%	15%	16%	28%	37%	22%	117%
Germany	2007	G08	481.5	390.0	23%	26%	8%	20%	18%	35%	23%	7%	49%	3%	15%	7%	48%	30%	13%	15%	22%	17%	18%	**146%**
Hungary	2003	G15	404.7	171.8	30%	14%	7%	13%	15%	23%	21%	8%	24%	2%	14%	6%	67%	36%	11%	10%	21%	18%	14%	86%
Ireland	2008	G15	323.3	142.6	20%	14%	6%	12%	13%	16%	18%	7%	23%	3%	27%	8%	53%	33%	9%	11%	18%	20%	15%	82%
Italy	2005	G10	538.6	197.3	33%	30%	11%	26%	23%	34%	44%	13%	36%	6%	44%	23%	112%	67%	21%	25%	28%	36%	25%	**146%**
Latvia	2011	G10	399.4	178.2	29%	16%	9%	14%	13%	31%	19%	8%	38%	2%	27%	7%	51%	26%	9%	11%	18%	25%	16%	107%
Netherlands	2007	G11	365.4	154.5	21%	14%	6%	12%	12%	16%	18%	6%	26%	3%	18%	8%	97%	34%	10%	11%	17%	25%	16%	100%
Netherlands	2010	G11	420.4	186.3	26%	17%	8%	14%	14%	21%	21%	7%	28%	3%	22%	9%	100%	36%	11%	12%	20%	28%	18%	110%
Portugal	2015	G15	334.3	142.3	20%	15%	7%	14%	14%	21%	24%	8%	29%	2%	22%	10%	31%	33%	9%	10%	19%	20%	14%	81%
Romania	2012	G15	567.5	221.8	37%	25%	10%	18%	20%	27%	32%	11%	35%	4%	39%	14%	55%	37%	13%	17%	26%	28%	21%	107%
Spain	2013	G08	439.2	186.5	29%	17%	7%	16%	16%	22%	29%	9%	33%	5%	16%	10%	64%	43%	13%	14%	20%	27%	18%	124%
Sweden	2010	G15	312.2	163.7	17%	12%	4%	10%	10%	13%	16%	5%	20%	1%	13%	6%	24%	30%	8%	10%	15%	15%	12%	89%
United Kingdom	2008	G07	357.5	171.8	28%	17%	6%	15%	17%	22%	25%	10%	40%	12%	23%	10%	64%	35%	11%	14%	24%	23%	18%	86%
Mean			*415.4*	*199.7*	*26%*	*18%*	*7%*	*15%*	*15%*	*23%*	*22%*	*8%*	*31%*	*4%*	*22%*	*9%*	*58%*	*36%*	*11%*	*12%*	*20%*	*23%*	*17%*	*105%*

Table S5. Single food contribution to nutrients RDAs

Calculated percentage fiber and nutrient contributions of main fruit, juice, vegetable, and legumes groups from all EFSA elderly studies to the sum from all fruit, juice, vegetables, and legumes.

Different food groups percentage contribution to total of all fiber and nutrients from F, J, L, V in all EFSA elderly studies	Dietary fiber (g/d) *	Potassium (mg/d, from g/d) *	Calcium (mg/d)*	Magnesium (mg/d)	Phosphorus (mg/d)	Iron (mg/d)	Copper (mg/d, from ug/d)	Zinc (mg/d)	Manganese (mg/d)*	Selenium (µg/d)	RAE (µg/d)	Vitamin E (mg/d)	Vitamin K1 (µg/d)*	Thiamin (mg/d)	Riboflavin (mg/d)	Niacin (mg/d)	Vitamin B6 (mg/d)	Folate (µg/d)	Pantothenate (mg/d)*	Vitamin C (mg/d)
Total of all F, J, L, V products in all EFSA elderly studies:	113.6	17385.5	1826.6	981.1	2163.9	37.9	4.2	12.9	11.6	40.7	3268.9	28.0	1089.7	8.2	2.6	36.7	6.3	1896.1	17.7	1655.2
All berries (except strawberries)	1.3%	0.7%	0.9%	0.8%	0.9%	1.6%	1.3%	1.2%	2.8%	0.0%	0.1%	1.9%	0.0%	0.3%	1.1%	0.7%	0.8%	0.6%	1.5%	1.8%
All citrus fruits excluding oranges	2.6%	1.6%	3.4%	2.0%	1.6%	0.7%	2.7%	0.4%	0.4%	0.3%	0.6%	1.6%	0.0%	4.0%	2.5%	2.3%	2.0%	2.2%	2.2%	5.6%
Apples (including dried and compote)	12.1%	6.7%	3.7%	5.5%	4.4%	2.9%	9.6%	0.0%	3.5%	0.0%	0.4%	3.9%	5.8%	4.9%	15.1%	3.7%	12.7%	0.0%	6.6%	4.2%
Apricots (dried and fresh and compote)	0.0%	0.8%	0.4%	0.5%	0.4%	0.8%	0.7%	0.4%	0.4%	1.2%	0.3%	0.0%	0.0%	0.1%	0.8%	0.6%	0.4%	0.1%	0.5%	0.1%
Avocados	0.0%	0.4%	0.1%	0.4%	0.3%	0.2%	1.4%	0.8%	0.3%	0.0%	0.0%	2.0%	0.0%	0.2%	1.2%	0.5%	0.9%	0.1%	1.1%	0.1%
Bananas	2.9%	4.4%	0.8%	6.3%	2.4%	1.6%	5.2%	2.8%	7.2%	0.0%	0.2%	1.3%	0.0%	4.0%	4.2%	5.0%	11.6%	1.7%	4.5%	1.1%
Canned or jarred fruit (excluding cherries)	0.1%	0.3%	0.2%	0.3%	0.2%	0.4%	0.4%	0.3%	0.9%	0.0%	0.1%	0.1%	0.0%	0.2%	0.2%	0.4%	0.3%	0.1%	0.2%	0.2%
Cherries (all, included canned-jarrred)	0.9%	0.7%	0.4%	0.5%	0.6%	0.5%	1.1%	0.4%	0.3%	0.0%	0.1%	0.3%	0.0%	0.2%	0.1%	0.3%	0.8%	0.1%	0.7%	0.1%
Dates (fresh and dried)	0.3%	0.2%	0.1%	0.2%	0.1%	0.2%	0.2%	0.1%	0.2%	0.3%	0.0%	0.1%	0.0%	0.0%	0.1%	0.2%	0.1%	0.0%	0.2%	0.0%
Dried fruit (excluding prunes, figs, dates, and apple)	0.3%	0.8%	0.6%	0.5%	0.7%	0.9%	1.4%	0.3%	0.4%	0.3%	0.0%	0.2%	0.0%	0.2%	0.1%	0.3%	0.6%	0.1%	0.1%	0.0%
Figs (fresh and dried)	0.0%	0.2%	0.4%	0.3%	0.1%	0.3%	0.3%	0.3%	0.2%	0.0%	0.0%	0.0%	0.0%	0.0%	0.1%	0.1%	0.2%	0.0%	0.1%	0.0%
Fruit from jams and marmalade	1.1%	0.6%	1.3%	0.7%	0.6%	1.0%	0.8%	0.6%	0.8%	0.4%	0.0%	0.1%	0.0%	0.1%	0.1%	0.1%	0.0%	0.1%	0.1%	0.6%
Grapes	1.4%	1.6%	0.7%	0.9%	1.1%	0.8%	2.7%	0.0%	0.7%	0.0%	0.0%	0.9%	0.0%	1.1%	0.5%	0.7%	0.8%	0.4%	0.9%	0.2%
Kaki and similar-	0.0%	0.3%	0.1%	0.2%	0.2%	0.1%	0.5%	0.2%	0.5%	0.0%	0.1%	0.0%	0.0%	0.1%	0.4%	0.2%	0.0%	0.1%	0.0%	0.2%
Kiwi	2.0%	1.4%	1.5%	1.1%	1.3%	0.5%	3.4%	0.7%	0.6%	0.0%	0.1%	6.5%	0.0%	0.0%	0.0%	0.7%	1.2%	1.5%	2.0%	3.6%
Mangoes	0.2%	0.1%	0.1%	0.1%	0.1%	0.1%	0.2%	0.1%	0.2%	0.0%	0.3%	0.6%	0.0%	0.1%	0.2%	0.4%	0.2%	0.2%	0.2%	0.3%
Miscellaneous fruits and mixed fruit products	1.3%	0.6%	0.5%	0.2%	0.2%	1.0%	0.4%	0.3%	0.1%	0.1%	0.1%	0.2%	0.0%	0.2%	0.3%	0.2%	0.2%	0.1%	0.1%	0.4%
Nectarines	0.4%	0.6%	0.1%	0.3%	0.4%	0.2%	0.8%	0.3%	0.3%	0.0%	0.1%	0.6%	0.1%	0.1%	0.3%	0.7%	0.2%	0.2%	0.8%	0.1%
Olives	0.0%	0.1%	0.5%	0.3%	0.1%	0.4%	0.7%	0.0%	0.0%	0.0%	0.1%	1.0%	0.0%	0.0%	0.0%	0.0%	0.0%	0.0%	0.0%	0.0%
Oranges only	4.3%	2.9%	5.4%	3.3%	3.0%	1.2%	2.9%	0.0%	0.7%	0.0%	0.1%	5.1%	0.0%	11.0%	4.7%	5.6%	3.3%	7.1%	6.3%	12.9%
Papayas	0.0%	0.0%	0.0%	0.0%	0.0%	0.0%	0.0%	0.0%	0.0%	0.0%	0.0%	0.0%	0.0%	0.0%	0.0%	0.0%	0.0%	0.0%	0.0%	0.1%
Peaches	0.0%	1.2%	0.5%	1.2%	1.3%	1.4%	1.8%	1.0%	1.1%	3.2%	0.4%	0.0%	0.7%	0.3%	2.0%	2.1%	0.4%	0.2%	1.2%	2.4%
Pears (including compote)	7.3%	1.9%	1.2%	1.6%	1.3%	1.1%	3.6%	2.4%	1.3%	0.0%	0.2%	0.0%	1.0%	1.1%	4.7%	1.7%	1.9%	1.0%	1.4%	0.6%

Pineapple (including compote)	0.3%	0.2%	0.2%	0.3%	0.1%	0.1%	0.6%	0.2%	3.5%	0.0%	0.0%	0.1%	0.0%	0.3%	0.0%	0.2%	0.4%	0.2%	0.2%	0.9%
Plums and dried prunes	0.4%	1.5%	0.9%	1.0%	1.1%	1.2%	2.3%	1.0%	1.0%	0.0%	0.8%	2.0%	0.5%	0.4%	0.8%	2.4%	0.5%	0.1%	0.6%	0.2%
Strawberries	4.7%	1.4%	1.3%	1.7%	1.7%	0.9%	1.0%	1.1%	3.8%	0.0%	0.0%	2.0%	0.4%	0.3%	1.1%	2.3%	0.7%	4.5%	3.0%	4.9%
All orange juice and nectar	0.0%	3.9%	2.7%	4.2%	3.1%	0.8%	2.9%	3.2%	1.1%	0.0%	0.5%	2.2%	0.0%	16.5%	3.2%	4.5%	2.6%	7.0%	4.4%	7.7%
Juice and nectar, others not already listed	2.2%	2.4%	1.3%	2.2%	1.6%	1.0%	1.5%	0.2%	0.9%	0.0%	0.0%	0.1%	0.0%	6.0%	2.4%	2.5%	3.0%	1.9%	2.2%	6.0%
Juice and nectar, peach and apricot	0.0%	0.0%	0.0%	0.0%	0.0%	0.1%	0.1%	0.0%	0.0%	0.1%	0.0%	0.0%	0.0%	0.0%	0.1%	0.1%	0.0%	0.0%	0.0%	0.1%
Juice and nectar, pear	0.0%	0.0%	0.0%	0.0%	0.0%	0.0%	0.0%	0.0%	0.0%	0.0%	0.0%	0.0%	0.0%	0.0%	0.0%	0.0%	0.0%	0.0%	0.0%	0.0%
Juice and nectar, pineapple	0.0%	0.0%	0.1%	0.1%	0.0%	0.1%	0.1%	0.1%	0.8%	0.0%	0.0%	0.0%	0.0%	0.1%	0.0%	0.0%	0.1%	0.1%	0.0%	0.1%
Juice, apples	0.0%	8.3%	4.3%	6.6%	3.9%	9.5%	0.0%	1.7%	9.7%	0.0%	0.0%	0.2%	0.0%	0.3%	1.7%	4.3%	2.5%	0.1%	2.8%	6.6%
Juice, carrot	0.0%	0.1%	0.1%	0.1%	0.1%	0.0%	0.1%	0.1%	0.1%	0.0%	1.3%	0.0%	0.0%	0.0%	0.2%	0.1%	0.2%	0.0%	0.1%	0.0%
Juice, grape	0.0%	0.0%	0.1%	0.1%	0.1%	0.3%	0.0%	0.1%	0.1%	0.3%	0.0%	0.0%	0.0%	0.0%	0.0%	0.0%	0.1%	0.0%	0.0%	0.0%
Juice, grapefruit	0.0%	0.2%	0.2%	0.2%	0.1%	0.2%	0.1%	0.0%	0.5%	0.7%	0.0%	0.2%	0.0%	0.1%	0.1%	0.2%	0.1%	0.1%	0.1%	0.5%
Juice, lemon and lime	0.0%	0.1%	0.1%	0.1%	0.1%	0.0%	0.1%	0.0%	0.0%	0.4%	0.0%	0.0%	0.0%	0.1%	0.1%	0.0%	0.1%	0.1%	0.1%	0.4%
Juice, tomato	0.0%	0.3%	0.1%	0.3%	0.2%	0.3%	0.4%	0.2%	0.2%	0.0%	0.1%	0.9%	0.0%	0.1%	0.2%	0.5%	0.2%	0.1%	0.3%	0.1%
Juices from berries	0.0%	0.1%	0.1%	0.0%	0.0%	0.1%	0.0%	0.1%	0.0%	0.0%	0.0%	0.0%	0.0%	0.0%	0.0%	0.0%	0.0%	0.0%	0.0%	0.3%
All legumes (excluding peas and lentils)	8.0%	4.0%	5.3%	8.8%	10.9%	8.9%	8.6%	12.2%	9.9%	20.4%	0.4%	3.9%	3.0%	3.2%	5.0%	3.5%	4.2%	6.3%	4.2%	0.4%
Lentils	2.9%	0.8%	0.3%	1.1%	3.0%	3.4%	3.2%	4.8%	2.1%	15.5%	0.0%	0.7%	0.0%	1.3%	1.1%	1.5%	0.9%	1.6%	1.4%	0.0%
All peas	4.2%	1.9%	1.5%	4.1%	6.7%	5.9%	1.2%	10.0%	4.3%	0.8%	0.8%	1.1%	3.1%	7.8%	2.5%	5.6%	1.7%	1.8%	2.0%	1.0%
Dried herbs	0.0%	0.2%	2.1%	0.7%	0.3%	4.3%	0.4%	0.8%	1.9%	0.0%	0.5%	0.0%	0.0%	0.0%	0.3%	0.3%	0.0%	0.0%	0.0%	0.1%
Fresh herbs	0.1%	0.8%	2.9%	1.0%	0.7%	4.2%	0.4%	1.8%	1.3%	0.3%	1.5%	0.5%	2.5%	0.2%	1.1%	0.6%	0.3%	0.6%	0.1%	0.8%
All leafy greens	0.1%	2.3%	6.3%	4.6%	1.7%	4.3%	0.6%	3.1%	3.4%	1.8%	13.2%	3.9%	23.3%	0.6%	2.6%	2.1%	1.8%	4.7%	1.0%	1.7%
All lettuces	2.2%	2.1%	2.2%	1.5%	1.7%	0.6%	1.2%	2.5%	2.0%	0.0%	0.3%	3.8%	19.4%	2.8%	3.1%	2.2%	0.5%	5.2%	1.8%	0.1%
All mushrooms	0.5%	1.4%	0.1%	0.7%	2.6%	0.8%	4.0%	3.0%	0.5%	24.1%	0.0%	0.1%	0.0%	0.9%	6.3%	4.4%	0.9%	1.2%	7.4%	0.0%
All onions	5.2%	2.2%	4.5%	2.3%	3.0%	2.4%	2.6%	2.3%	2.4%	0.0%	0.2%	2.8%	0.0%	3.6%	2.2%	2.3%	4.4%	1.7%	0.6%	0.6%
Tomato puree	0.9%	1.7%	0.6%	1.3%	1.0%	0.9%	2.2%	1.1%	0.7%	0.6%	1.0%	4.3%	0.0%	2.7%	1.1%	2.3%	1.0%	0.5%	0.1%	0.6%
All tomatoes	7.8%	11.8%	4.1%	8.1%	8.7%	6.1%	9.1%	7.4%	8.0%	1.1%	8.1%	20.8%	3.5%	9.2%	4.9%	15.3%	8.2%	8.8%	10.8%	9.8%
All types of cabbage	4.9%	2.5%	5.6%	2.2%	2.4%	2.0%	1.2%	2.9%	2.2%	4.7%	0.2%	0.4%	12.2%	4.8%	1.4%	1.7%	3.5%	7.3%	2.4%	5.2%
Asparagus	0.2%	0.3%	0.2%	0.2%	0.6%	0.2%	0.5%	0.7%	0.2%	4.2%	0.0%	1.0%	0.0%	0.2%	1.0%	0.8%	0.4%	1.0%	0.3%	0.1%
Aubergines	0.6%	0.3%	0.2%	0.4%	0.4%	0.2%	0.3%	0.3%	0.5%	0.0%	0.0%	0.0%	0.0%	0.0%	0.2%	0.1%	0.2%	0.1%	0.1%	0.0%
Beetroot	1.2%	0.9%	0.8%	0.8%	0.6%	0.5%	1.1%	1.2%	1.3%	0.0%	0.0%	0.0%	0.0%	0.0%	0.0%	0.3%	0.5%	2.5%	0.5%	0.0%
Broccoli	1.9%	0.9%	1.4%	1.1%	2.1%	1.2%	0.9%	2.3%	1.3%	1.8%	1.2%	4.5%	9.3%	0.4%	1.7%	1.0%	1.3%	1.3%	1.2%	2.0%
Brussels sprouts	0.6%	0.5%	0.3%	0.4%	0.7%	0.3%	0.2%	0.5%	0.4%	0.5%	0.1%	0.2%	2.4%	0.1%	0.6%	0.2%	0.8%	1.4%	0.5%	0.5%
Carrots	0.0%	5.1%	6.9%	3.4%	4.3%	3.9%	1.7%	5.7%	3.2%	9.1%	57.7%	7.4%	3.1%	1.8%	2.8%	2.0%	4.1%	5.5%	5.2%	0.9%
Cauliflowers	1.4%	1.0%	0.9%	1.0%	1.7%	1.0%	0.6%	1.3%	1.0%	2.0%	0.0%	0.3%	2.2%	0.9%	1.0%	0.9%	2.0%	2.1%	2.2%	1.5%

Celeriacs	0.0%	0.2%	0.2%	0.1%	0.2%	0.2%	0.1%	0.1%	0.1%	0.0%	0.0%	0.0%	0.0%	0.1%	0.1%	0.0%	0.1%	0.2%	0.0%	0.0%
Celery (including leaves)	0.4%	0.6%	0.8%	0.2%	0.3%	0.1%	0.1%	0.3%	0.2%	0.0%	0.0%	0.1%	0.2%	0.1%	0.3%	0.3%	0.2%	0.2%	0.6%	0.0%
Courgettes	0.4%	1.7%	1.3%	2.2%	1.6%	1.0%	1.7%	2.1%	0.8%	2.2%	0.5%	1.0%	0.3%	0.3%	0.7%	0.0%	1.0%	1.6%	0.9%	0.3%
Cucumbers	1.0%	1.5%	1.9%	1.7%	1.8%	1.3%	1.2%	1.3%	1.3%	0.0%	0.3%	0.2%	3.2%	0.6%	1.3%	0.9%	0.3%	1.2%	3.0%	0.2%
Garlic	0.0%	0.3%	0.1%	0.2%	0.8%	0.5%	0.1%	0.7%	0.4%	0.5%	0.0%	0.0%	0.0%	0.2%	0.1%	0.1%	0.6%	0.0%	0.0%	0.1%
Leeks	1.8%	1.0%	2.4%	0.5%	1.1%	0.6%	0.7%	0.8%	0.6%	0.0%	0.1%	1.1%	0.9%	0.6%	0.8%	1.2%	1.3%	2.5%	1.0%	0.1%
Melon and watermelon	1.0%	1.3%	0.9%	1.4%	0.7%	1.0%	0.6%	1.6%	0.1%	0.0%	0.2%	0.5%	0.0%	0.9%	0.6%	1.4%	2.6%	0.4%	1.6%	1.1%
Miscellaneous vegetables (including canned or jarred)	3.6%	3.6%	9.0%	2.9%	4.0%	9.8%	2.1%	5.3%	3.1%	1.9%	5.9%	1.4%	2.5%	1.7%	5.6%	2.4%	3.1%	5.0%	3.1%	1.7%
Parsnip roots and parsley root	0.2%	0.4%	0.6%	0.3%	0.5%	0.2%	0.2%	0.4%	0.4%	0.4%	0.0%	0.5%	0.0%	0.2%	0.2%	0.3%	0.4%	0.3%	0.3%	0.2%
Rhubarbs	0.1%	0.1%	0.3%	0.1%	0.1%	0.0%	0.1%	0.1%	0.1%	0.0%	0.0%	0.1%	0.0%	0.0%	0.1%	0.0%	0.0%	0.0%	0.0%	0.0%
Squashes and pumpkin	0.0%	0.3%	0.6%	0.5%	0.4%	0.4%	0.3%	0.6%	0.4%	0.0%	0.4%	1.5%	0.0%	0.7%	0.1%	0.2%	0.2%	0.2%	0.9%	0.3%
Swede and turnip	0.4%	0.4%	0.8%	0.3%	0.6%	0.2%	0.1%	0.3%	0.3%	0.5%	0.0%	0.0%	0.0%	0.1%	0.1%	0.4%	0.4%	0.3%	0.3%	0.3%
Sweet corn (including canned)	0.2%	0.2%	0.0%	0.4%	0.5%	0.2%	0.1%	0.5%	0.2%	0.0%	0.0%	0.3%	0.0%	0.4%	0.3%	0.8%	0.2%	0.3%	0.4%	0.1%
Sweet peppers, peppers, chilli peppers	2.0%	1.3%	0.4%	1.2%	1.1%	1.1%	1.3%	1.6%	1.1%	0.0%	1.4%	3.5%	0.1%	0.9%	2.4%	1.5%	3.8%	4.1%	1.6%	7.9%

Table S6. Percentage contribution to U.S. RDA or AI for males >70 years

Mean of European G clusters G07, G08, G10, G11, and G15 availability of fruit, vegetables, and legumes and associated nutrients (as percentage contribution to U.S. RDA or AI for males >70 years), mean of elderly EFSA studies, and the difference between mean European availability and mean consumption for elderly.

Country EFSA database	G cluster	Consumption total F+V+J+L mean (g/p/d)	Energy (kcal/d)	Dietary fiber (g/d) *	Potassium (mg/d) *	Calcium (mg/d)*	Magnesium (mg/d)	Phosphorus (mg/d)	Iron (mg/d)	Copper (mg/d)	Zinc (mg/d)	Manganese (mg/d)*	Selenium (µg/d)	RAE (µg/d)	Vitamin E (mg/d)	Vitamin K1 (µg/d)*	Thiamin (mg/d)	Riboflavin (mg/d)	Niacin (mg/d)	Vitamin B6 (mg/d)	Folate (µg/d)	Pantothenate (mg/d)*	Vitamin C (mg/d)
U.S. RDAs (AI) males >70 y*				*30**	*4700**	*1200**	*420*	*700*	*8*	*0.9*	*11*	*2.3**	*55*	*900*	*15*	*120**	*1.2*	*1.3*	*16*	*1.7*	*400*	*5**	*90*
Availability	G07	584.3	202.1	26%	24%	10%	15%	23%	30%	29%	10%	34%	5%	31%	11%	70%	50%	14%	19%	26%	36%	29%	134%
Availability	G08	588.2	198.3	26%	25%	11%	15%	22%	28%	34%	9%	34%	5%	31%	10%	97%	41%	13%	15%	29%	38%	28%	132%
Availability	G10	626.2	277.3	38%	28%	19%	22%	36%	53%	38%	15%	42%	6%	26%	12%	99%	51%	15%	18%	27%	44%	28%	141%
Availability	G11	652.8	212.2	28%	27%	11%	16%	24%	31%	34%	11%	44%	7%	47%	11%	70%	43%	16%	17%	28%	41%	34%	132%
Availability	G15	620.1	222.3	33%	27%	12%	17%	26%	32%	33%	10%	36%	5%	31%	13%	104%	52%	14%	18%	32%	42%	29%	184%
Mean availability EU clusters		*614.3*	*222.4*	*30%*	*26%*	*13%*	*17%*	*26%*	*35%*	*34%*	*11%*	*38%*	*6%*	*33%*	*11%*	*88%*	*47%*	*15%*	*17%*	*28%*	*40%*	*30%*	*145%*
Mean male elderly EFSA		415.4	200	18%	18%	7%	11%	15%	23%	22%	6%	24%	4%	17%	9%	43%	33%	10%	11%	18%	23%	17%	88%
Mean availability - mean elderly		*198.9*	*22.7*	*12%*	*9%*	*5%*	*6%*	*11%*	*12%*	*11%*	*5%*	*14%*	*2%*	*16%*	*3%*	*45%*	*15%*	*5%*	*6%*	*11%*	*18%*	*13%*	*57%*
U.S. RDAs (AI) females >70 y*				*30**	*4700**	*1200**	*420*	*700*	*8*	*0.9*	*11*	*2.3**	*55*	*900*	*15*	*120**	*1.2*	*1.3*	*16*	*1.7*	*400*	*5**	*90*
Availability	G07	584.3	202.1	38%	24%	10%	20%	23%	30%	29%	13%	43%	5%	40%	11%	93%	55%	16%	21%	30%	36%	29%	161%
Availability	G08	588.2	198.3	37%	25%	11%	20%	22%	28%	34%	12%	44%	5%	40%	10%	129%	45%	16%	18%	32%	38%	28%	158%
Availability	G10	626.2	277.3	54%	28%	19%	28%	36%	53%	38%	21%	54%	6%	34%	12%	132%	56%	18%	21%	31%	44%	28%	169%
Availability	G11	652.8	212.2	41%	27%	11%	21%	24%	31%	34%	15%	56%	7%	61%	11%	94%	47%	19%	19%	32%	41%	34%	158%
Availability	G15	620.1	222.3	47%	27%	12%	23%	26%	32%	33%	14%	46%	5%	40%	13%	139%	56%	17%	20%	36%	42%	29%	221%
Mean availability EU clusters		*614.3*	*222.4*	*43%*	*26%*	*13%*	*22%*	*26%*	*35%*	*34%*	*15%*	*49%*	*6%*	*43%*	*11%*	*117%*	*52%*	*17%*	*20%*	*32%*	*40%*	*30%*	*173%*
Mean female elderly EFSA		415.4	200	26%	18%	7%	15%	15%	23%	22%	8%	31%	4%	22%	9%	58%	36%	11%	12%	20%	23%	17%	105%
Mean availability - mean elderly		*198.9*	*22.7*	*18%*	*9%*	*5%*	*8%*	*11%*	*12%*	*11%*	*8%*	*18%*	*2%*	*21%*	*3%*	*60%*	*16%*	*6%*	*7%*	*12%*	*18%*	*13%*	*68%*

Table S7. Trace plots of the Bayesian multilevel hierarchical model

The trace plots have shown the convergence of the Bayesian multilevel hierarchical model; no patterns have been revealed in MCMC iteration across chains

Overall fruit and vegetable intake	Vegetables and vegetable products
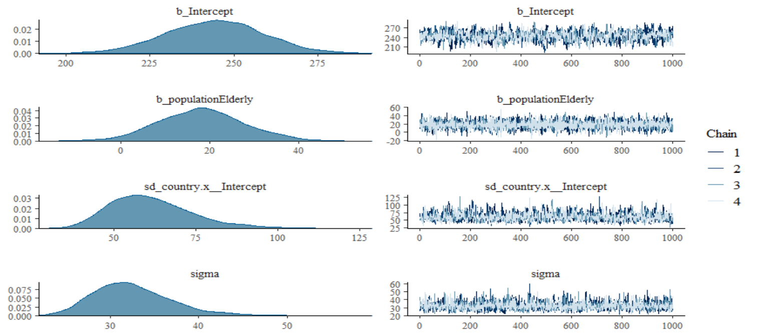	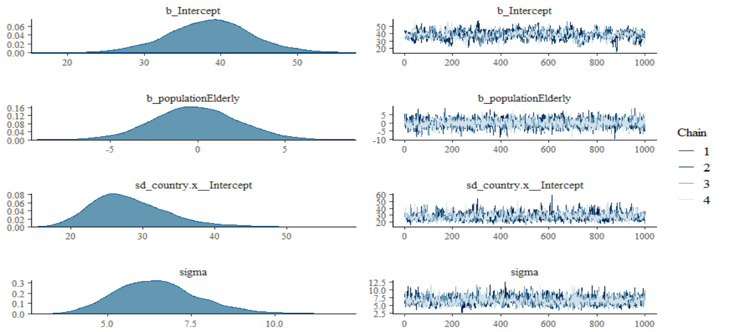
**Legumes, nuts, oilseeds and spices**	**Fruit and fruit products**
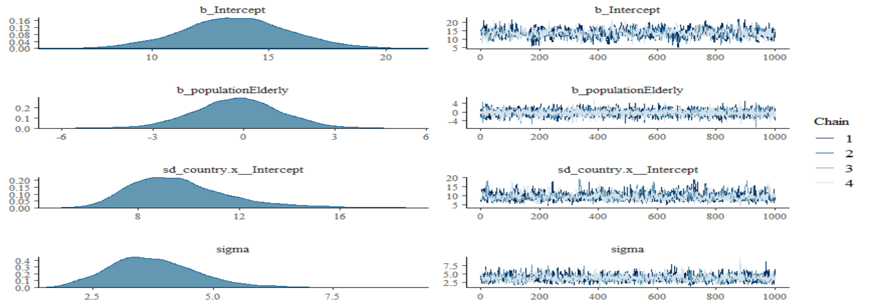	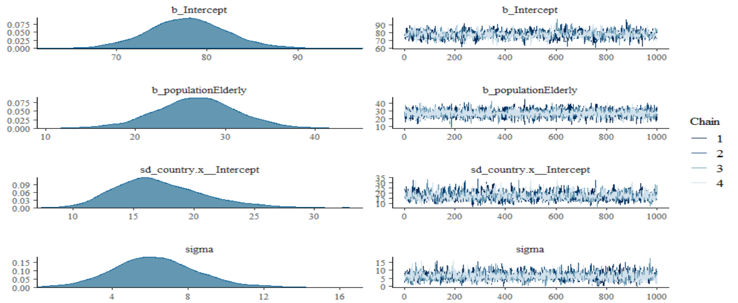
**Fruit and vegetable juices and nectars (including concentrates)**	

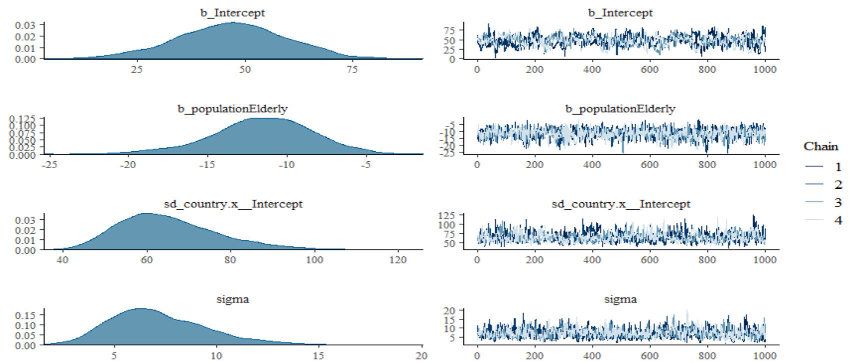
b_Intercept
b_populationElderly
sd_country.x__Intercept
sigma
Chain
1
2
3
4

THE ROUND website

WoRld map of cOnsUmption of fruit and vegetables and Nutrient Deficits (ROUND) project

Data presented in this paper are extracted from the website www.round-project.com. The ROUND project (www.round-project.com) is an ongoing collaborative effort to develop a dynamic world map showing fruit and vegetables consumption in different countries and regions, and to determine the percentage contribution of different nutrients from fruits, vegetables and legumes, compared to recommended daily nutrient intakes. The ROUND project is developed by the University of Padua and Zeta Research Srl, and it is supported and conducted in collaboration with Juice Plus+ Science Institute (JPSI).

At the moment the maps on the website www.round-project.com show availability data (mean g/capita/day) for 159 fruit, vegetable, legume, and starchy roots/tuber products for 179 countries, and consumption data for Europe, USA, Australia, Argentina, Bangladesh, Plurinational State of Bolivia, Burkina Faso, Guatemala, India, Kenya, Pakistan, the Democratic Republic of the Congo, the Lao People's Democratic Republic, Uganda and Zambia for the following food groups: fruit and fruit products, vegetables and vegetable products; fruit and vegetable juices and nectars (including concentrates); starchy roots or tubers and products thereof, sugar plants.

In the consumption map is it possible to choose a food sub-group and a Population Age and see over the years 1998 to 2017 consumption range (g/capita/day) and see how the coloration of the countries changes: green indicates countries in a mean consumption of the selected food sub-group, red indicates countries under selected consumption range, and orange countries above. Near range limits color is lighter. Grey indicates countries with no data for those years.

In the availability map is it possible to choose a Region and a food sub-group and select an availability range (g/capita/day) and see how the coloration of the countries changes: green indicates countries with a mean availability of the selected food sub-group, red indicates countries under selected availability range, and orange countries above. Near range limits color is lighter. Grey indicates countries for which IEDI GEMs do not hold data in their database.
